# Anatomical predictors of gastrostomy tube placement after large vessel occlusion ischemic stroke

**DOI:** 10.3389/fneur.2025.1611736

**Published:** 2025-09-18

**Authors:** Margy McCullough-Hicks, Salman Ikramuddin, Soren Christensen, Michael Mlynash, Gregory Albers

**Affiliations:** ^1^Department of Neurology, University of Minnesota, Minneapolis, MN, United States; ^2^Department of Neurology, University of Texas Health Science Center at Houston, Houston, TX, United States; ^3^Department of Neurology, Stanford University, Palo Alto, CA, United States

**Keywords:** stroke, large vessel occlusion (LVO), gastrostomy (PEG), topography, voxel based lesion symptom mapping

## Abstract

Dysphagia is a common neurologic deficit following ischemic stroke. As a result, patients often require percutaneous endoscopic gastrostomy (PEG) tube placement for safe maintenance of sufficient caloric intake. Right hemispheric strokes have previously been associated with post-stroke dysphagia. We examined whether topographic location of post-large vessel occlusion (LVO) stroke MR diffusion-weighted imaging (DWI) lesions associated with the need for placement of PEG tube. A retrospective registry of 898 patients evaluated for acute treatment of suspected LVO stroke was used. Sixty-five patients underwent post-stroke PEG placement, and 65 additional patients were selected as propensity matches based on age, baseline NIH Stroke Scale score, and recanalization status. Binary masks of 24–72-h post-stroke DWI lesions were co-registered to standard template space. Voxel-based lesion symptom mapping (V2.55), rewritten to perform logistic regression at each voxel, was used to generate statistical maps of lesion contribution to PEG placement. Results: Uncorrected t-statistic maps demonstrated voxels in the right frontal, parietal, and temporal regions were associated with post-stroke PEG placement. Upon controlling for age and/or recanalization status, lesions in the right parietal lobe were associated with need for PEG. After controlling for lesion volume, this association weakened. After controlling for all variables, there were no topographical regions associated with PEG placement. There does not appear to be any topographic region on post-stroke diffusion MRI that is significantly associated with need for PEG placement in patients with LVO stroke after controlling for age, recanalization status, and lesion size.

## Introduction

Nearly 800,000 acute ischemic strokes occur in the United States annually, and are a leading cause of disability (1). High rates of impairment following stroke are in part mediated by post-stroke dysphagia (PSD), which can occur in as many as 78% of patients (2). Patients with dysphagia suffer a higher risk of malnutrition and aspiration pneumonia during the period of stroke recovery (3–5). Furthermore, PSD is associated with increased rates of mortality and negative effects on quality-of-life following stroke (6). Placement of percutaneous endoscopic gastrostomy (PEG) tube is a mainstay of clinical management to mitigate the risk of aspiration while recovery of swallowing functions occurs. Disease modifying treatment options are limited (7).

PEG dependence is simultaneously seen as a key component in quality-of-life discussion with patients and families, as it represents a significant deviation from most patients’ baseline capacity for nutritional intake. As such, ability to predict the need for PEG placement in patients with ischemic stroke is needed to help guide acute treatment decisions, conduct goals of care discussions, and successfully individualize patient care. There is limited data to assist clinicians in the prediction of PEG dependence in the literature today.

Previous efforts have been made to predict the risk of aspiration after ischemic stroke, demonstrating that lesions of the insular cortex and internal capsule were associated with post-stroke aspiration (8). To understand PSD more directly, one study utilized lesion symptom mapping to in patients with dysphagia to identify regions of interest that were associated with specific deficits in swallow function (9). This study demonstrated that, after controlling for age and lesion volume, right hemispheric lesions involving sensory-motor integration areas were associated with post-stroke dysphagia (9). Additional evidence supports controlling for age, stroke severity, and the success of acute stroke treatment such as recanalization status (10, 11).

We sought to utilize an MRI derived voxel-based lesion symptom mapping technique to determine the relationship between stroke location and subsequent need for PEG placement in a propensity matched cohort of patients evaluated for LVO stroke.

## Methods

We used a single-institution retrospective registry of 898 patients evaluated for acute treatment of suspected LVO ischemic stroke. Sixty-five cases within the cohort underwent post-stroke PEG tube placement. We performed propensity matching in another 65 patients. Cases and controls were matched on propensity scoring for distance metric minimizing difference between the logits of the propensity scores with a greedy nearest neighbor matching method in random order and a control to treated ratio of 1:5.

Matching was performed in SAS 9.4. Matching was based on age, baseline NIH stroke scale (NIHSS), and recanalization status. The match with the closest propensity score to each PEG case was included in the controls. Patients who declined PEG placement as part of goals of care planning were excluded from both cases and controls. In total, discussion around PEG placement was at least raised but not pursued in 154 patients due to goals-of-care decisions, often a transition to comfort measures. These patients were excluded because the goals-of-care decisions interrupted the usual relationship between dysphagia severity and PEG placement, making them a distinct subgroup for whom lesion-PEG associations could not be validly assessed. PEG placement at our institution depends on a multi-disciplinary evaluation of patient nutritional and swallowing status; it typically involves the neurology, speech therapy, nutrition, and procedural teams as well as multiple goals of care discussions with patients and/or their proxy decision-makers. PEG placement is generally pursued when a patient is otherwise ready for discharge from the hospital but haver persistent inability to consume sufficient nutrition safely by mouth.

Diffusion weighted brain MRI performed 24–72 h after stroke onset (and, when applicable, after acute stroke treatment) were utilized for lesion mapping. Binary masks of DWI lesions were created by two authors (MMH, SI). Lesions were co-registered to standard Montreal Neurological Institute (MNI) template space. Voxel-based lesion symptom mapping (V2.55) rewritten to perform logistic regression at each voxel was used to generate statistical maps of lesion contribution to PEG placement. Maps were thresholded to *p* < 0.01 on basis of cluster size and permutation method. Z-statistic maps were generated over voxels associated with the need for PEG placement. Additional maps were then generated controlling for age, lesion size, and/or recanalization status.

## Results

Sixty-five patients with post-stroke PEG placement and 65 propensity matched controls had sufficient data and neuroimaging for study inclusion and were analyzed. Baseline characteristics were similar between the groups in terms of age, sex, baseline NIHSS, and recanalization status (Table 1 contains baseline demographics). In the PEG tube cohort, mean (SD) age was 69.6 (13.5), baseline (when applicable, pre-treatment) NIHSS was 19.1 (7.0), 49.2% were female, 43.0% received IV thrombolysis, and 33.8% underwent EVT. In the propensity matched control cohort, mean (SD) age was 67.4 (16.8), baseline NIHSS was 19.4 (6.4), 50.7% were female, 46.1% received IV thrombolysis, and 33.8% underwent endovascular therapy (EVT).

**Table 1:** Baseline patient demographics.

PEG	PEG (*n* = 65)	Matched controls (*n* = 65)
Mean (SD) age	69.6 (13.5)	67.4 (16.8)
% Female	49.2%	50.7%
Mean (SD) NIHSS	19.1 (7.0)	19.4 (6.4)
% IV thrombolysis	43.0%	46.1%
% EVT (%TICI 2b-3)	33.8% (79%)	33.8% (79%)

**Figure 1:**
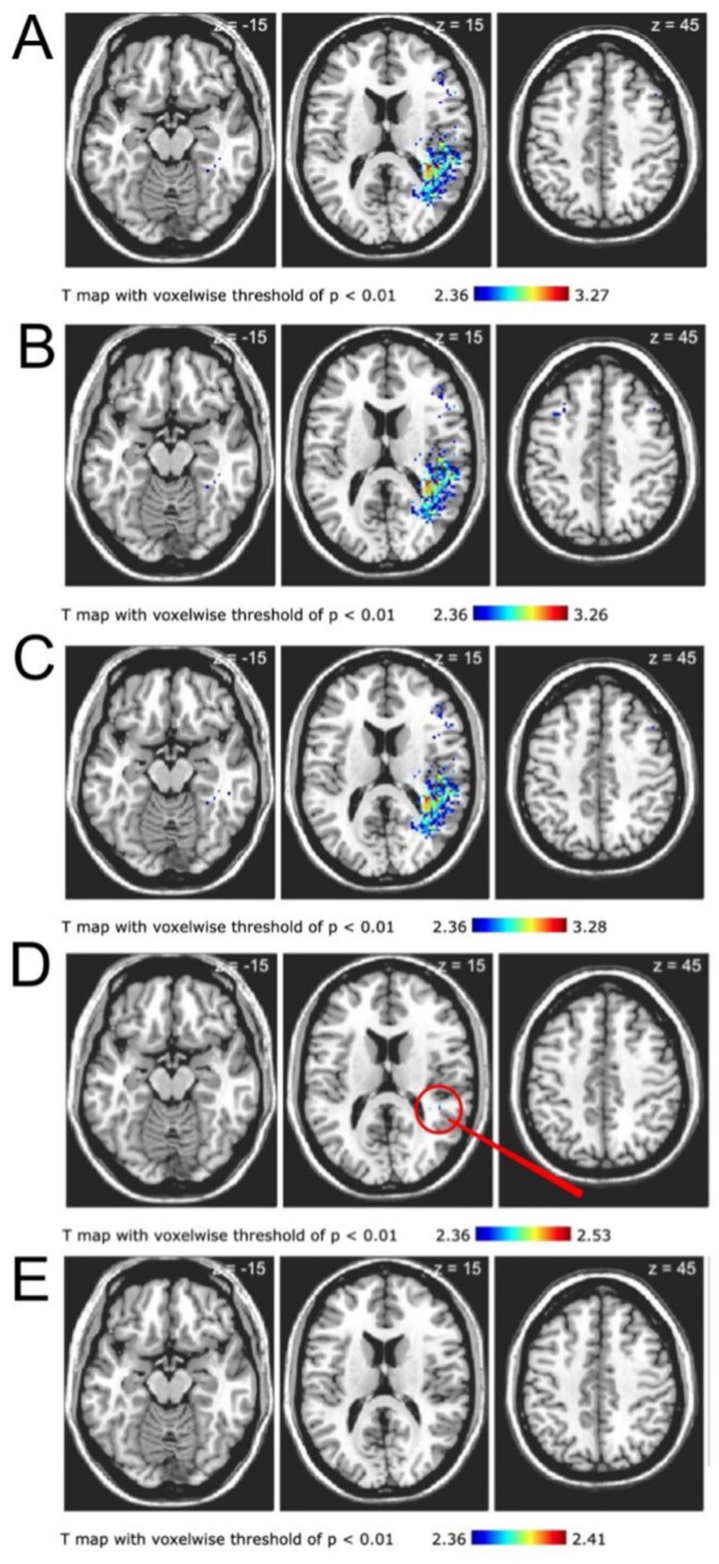
Neuroanatomic regions associated with need for post-stroke PEG placement. All images are T-maps with voxel threshold of *p* < 0.01 derived from post-stroke DWI sequences. Lesion maps are in neuroanatomic (not radiologic) space; statistically significant voxels are in the right hemisphere. **(A)** T-map depicts voxels in right parietal, frontal, and temporal regions associated with PEG placement. **(B)** T map after correcting for patient age depicts right parietal, frontal, and temporal regions associated with PEG placement. **(C)** T-map after correcting for recanalization status depicts voxels in right parietal, right frontal, and right temporal regions associated with PEG placement. **(D)** T-map after correcting for lesion volume depicts only two punctate areas of the right parietal lobe associated with PEG placement. Red arrow marks the regions of interest. **(E)** T-map after correcting for all variables (age, recanalization, and lesion size) shows no region associated with PEG placement. The differences between **A**, **B**, and **D** compared with **C** and **E** demonstrate the importance of lesion volume in the need for post-stroke PEG placement.

Voxel-based lesion symptom maps were generated using all post-stroke DWI lesions from 130 PEG cases and matched controls. Clusters of voxels, thresholded to a t-statistic of <0.01, in the right frontal, temporal and parietal lobes were associated with need for post-stroke PEG placement (Figure 1, panel A). After controlling for patient age there remained a similar relationship between right parietal infarct and PEG placement (Figure 1, panel B). Lesion symptom maps were also similar after controlling for recanalization status (in patients who underwent EVT, defined as Thrombolysis in Cerebral Infarction score) (Figure 1, panel C). However, after controlling for lesion size, only two punctate areas in the right parietal lobe were associated with PEG placement (Figure 1, panel D). Once all covariates were controlled for together (age, lesion size, and recanalization status), there was no topographic region that was associated with need for PEG placement in patients with LVO stroke (Figure 1, panel E).

## Discussion

Post-stroke dysphagia is a significant contributor to overall post-stroke disability. Little data exists to help practitioners predict the need for post-stroke PEG placement early in patients’ clinical courses. It is common for patients to wait days to weeks after incident stroke for determination of the need for PEG placement, prolonging hospital admissions and delaying goals of care discussions. Early PEG tube placement is associated with shorter length of stay without any increase in complications (12). We sought to test the hypothesis that post-stroke ischemic lesion location might predict the need for PEG placement. Utilizing voxel-based lesion mapping on 24–72-h post-stroke diffusion MRIs, we found that lesions in the right parietal lobe were associated with the need for post-stroke PEG placement, even after controlling for age and vessel recanalization status. However, after controlling for lesion volume, only two punctate regions of the right parietal lobe were associated with post-stroke PEG placement. Once for all covariates were controlled for, no topographic region was associated with the need for PEG following large vessel occlusion stroke.

Our data do not support the concept of a unique region of the brain independently associated with the need for post-stroke PEG placement. However, our sample size was limited. In uncontrolled analyses, clusters of voxels in the right parietal lobe were associated with PEG tube placement. This is consistent with previous publications. Wilmskotter et al. (9) conducted modified barium swallow studies on 68 patients, also using a voxel-based approached to assess the relationship between brain lesion location and 17 different aspects of swallow function. The study ultimately found that abnormal penetration aspiration scores were associated with voxels in the right precentral gyrus as well as the right post-central gyrus. Right parietal stroke was also associated with pharyngeal residue, impaired laryngeal elevation as well as impaired laryngeal vestibular closure. This was true even after controlling for age and lesion volume. While right hemispheric lesions were strongly associated with impaired swallow function, it must be noted that not all mechanical swallow dysfunction was associated with right hemispheric lesions. Impaired anterior hyoid excursion was associated with left hemispheric lesions.

Despite this, there is now a body of work that associates right hemispheric lesions to post-stroke dysphagia (13–18). In a separate study conducted by Crisan et al. (19) of 1,300 patients, younger age and left hemispheric stroke were more predictive of functional recovery of swallowing after PEG tube placement as compared to right hemispheric stroke. Importantly, the associated stroke lesion topography for PEG tube placement may differ from that of laryngeal dysfunction or other mechanical changes in swallow function. Our study remains unique in that it utilizes PEG tube placement at discharge as a primary outcome. If this primary outcome is utilized in future studies, we hope that it could enhance the clinical utility of the findings. Our data combined with preexisting literature may indicate that infarcts in the right hemisphere, particularly the right parietal lobe, could be implicated in post-stroke dysphagia.

There is a need for additional studies with larger sample size that also account for important clinical covariates, including non-neurological comorbidities such as malnutrition and frailty scores.

Stroke lesion volume appeared to be a potent covariate in our dataset; it has also previously been shown to contribute to post-stroke dysphagia (20). While prior studies indicate that stroke location, treatment status, patient age, and lesion volume all contribute to post-stroke dysphagia (9–11, 21), the relative weight of these factors as predictive variables is unclear. In this study, the loss of significance after specifically controlling for lesion volume could indicate its outsized role in post-stroke dysphagia. Indeed, swallowing dysfunction has also been described as a more diffuse, network-based neurological function that could be disrupted by all large strokes (18). Future studies investigating the strength of stroke volume alone as a primary predictor of post-stroke PEG placement are needed.

Our study was subject to limitations. First, it was conducted at a single center using a retrospective registry of only LVO strokes and institutional practices around PEG placement may not be uniform, which limits generalizability. Second, although our cohort included 898 patients evaluated for potential LVO stroke, only 65 underwent PEG placement (7.2%). This rate is higher than the ~3–5% prevalence reported in large unselected stroke cohorts (22), likely reflecting both enrichment for severe LVO strokes and the exclusion of 154 patients in whom PEG was discussed but not pursued. Excluding this subgroup reduced sample size but allowed us to focus on patients in whom lesion topography could be validly related to definitive PEG placement. Third, our modest sample size and inclusion criteria (restricted to patients with MRI within 24-72 h after stroke onset) further limit generalizability. Sample sizes greater than 90 subjects may generate more robust VLSM results, and increasingly larger samples sizes are being called for in VSLM research (23). Fourth, we used 1:1 matching between cases and controls. While larger control-to-case ratios (e.g., 1:2) can increase sample size, prior work has shown that gains in statistical efficiency diminish rapidly beyond 1:1 or 1:2 (24), and the substantial effort required for manual neuroimaging processing made 1:1 the best approach in balancing rigor and practicality. Future work with larger datasets should consider 1:2 matching. Finally, our study was limited by its outcome measures. We examined PEG placement as a binary outcome and were unable to evaluate specific mechanical features of dysphagia, the precise timing of PEG placement, or long-term feeding outcomes. Institutional practice during this study period was generally to consider PEG at 10–14 days post-stroke in patients with persistent dysphagia, consistent with guideline recommendations. Future prospective studies should collect granular timing data, assess early PEG removal and return to oral feeding, and evaluate whether lesion topography is associated with longer-term functional recovery.

## Data Availability

The raw data supporting the conclusions of this article will be made available by the authors, without undue reservation.

## References

[1] Feigin VL Brainin M Norrving B Martins S Sacco RL Hacke W. World stroke organization (WSO): global stroke fact sheet 2022. Int J Stroke. (2022) 17:18–29. doi: 10.1177/17474930211065917, PMID: 34986727

[2] Martino R Foley N Bhogal S Diamant N Speechley M Teasell R. Dysphagia after stroke: incidence, diagnosis, and pulmonary complications. Stroke. (2005) 36:2756–63. doi: 10.1161/01.STR.0000190056.76543.eb, PMID: 16269630

[3] Smithard DG O'Neill PA Parks C Morris J. Complications and outcome after acute stroke. Does dysphagia matter? Stroke. (1996) 27:1200–4. doi: 10.1161/01.str.27.7.1200, PMID: 8685928

[4] Chang MC Choo YJ Seo KC Yang S. The relationship between dysphagia and pneumonia in acute stroke patients: a systematic review and Meta-analysis. Front Neurol. (2022) 13:13. doi: 10.3389/fneur.2022.834240, PMID: 35370927 PMC8970315

[5] González-Fernández M Ottenstein L Atanelov L Christian AB. Dysphagia after stroke: an overview. Curr Phys Med Rehabil Rep. (2013) 1:187–96. doi: 10.1007/s40141-013-0017-y, PMID: 24977109 PMC4066736

[6] Ekberg O Hamdy S Woisard V Wuttge-Hannig A Ortega P. Social and psychological burden of dysphagia: its impact on diagnosis and treatment. Dysphagia. (2002) 17:139–46. doi: 10.1007/s00455-001-0113-5, PMID: 11956839

[7] Balcerak P Corbiere S Zubal R Kägi G. Post-stroke dysphagia: prognosis and treatment–a systematic review of RCT on interventional treatments for dysphagia following subacute stroke. Front Neurol. (2022) 13:3189. doi: 10.3389/fneur.2022.823189, PMID: 35547370 PMC9082350

[8] Galovic M Leisi N Müller M Weber J Abela E Kägi G. Lesion location predicts transient and extended risk of aspiration after supratentorial ischemic stroke. Stroke. (2013) 44:2760–7. doi: 10.1161/STROKEAHA.113.001690 23887840

[9] Wilmskoetter J Bonilha L Martin-Harris B Elm JJ Horn J Bonilha HS. Mapping acute lesion locations to physiological swallow impairments after stroke. Neuroimage Clin. (2019) 22:101685. doi: 10.1016/j.nicl.2019.101685, PMID: 30711683 PMC6357850

[10] Kumar S Langmore S Goddeau RP Jr Alhazzani A Selim M Caplan LR. Predictors of percutaneous endoscopic gastrostomy tube placement in patients with severe dysphagia from an acute-subacute hemispheric infarction. J Stroke Cerebrovasc Dis. (2012) 21:114–20. doi: 10.1016/j.jstrokecerebrovasdis.2010.05.010, PMID: 20851628 PMC3172374

[11] Broadley S Croser D Cottrell J Creevy M Teo E Yiu D. Predictors of prolonged dysphagia following acute stroke. J Clin Neurosci. (2003) 10:300–5. doi: 10.1016/S0967-5868(03)00022-5, PMID: 12763332

[12] Reddy KM Lee P Gor PJ Cheesman A Al-Hammadi N Westrich DJ. Timing of percutaneous endoscopic gastrostomy tube placement in post-stroke patients does not impact mortality, complications, or outcomes. World J Gastrointest Pharmacol Ther. (2022) 13:77–87. doi: 10.4292/wjgpt.v13.i5.77 36157266 PMC9453443

[13] Daniels SK Foundas AL Iglesia GC Sullivan MA. Lesion site in unilateral stroke patients with dysphagia. J Stroke Cerebrovasc Dis. (1996) 6:30–4. doi: 10.1016/S1052-3057(96)80023-1, PMID: 17894962

[14] May NH Pisegna JM Marchina S Langmore SE Kumar S Pearson WG Jr. Pharyngeal swallowing mechanics secondary to hemispheric stroke. J Stroke Cerebrovasc Dis. (2017) 26:952–61. doi: 10.1016/j.jstrokecerebrovasdis.2016.11.001, PMID: 27913200 PMC5409864

[15] Robbins J Levine RL Maser A Rosenbek JC Kempster GB. Swallowing after unilateral stroke of the cerebral cortex. Arch Phys Med Rehabil. (1993) 74:1295–300. doi: 10.1016/0003-9993(93)90082-L, PMID: 8259895

[16] Suntrup S Kemmling A Warnecke T Hamacher C Oelenberg S Niederstadt T. The impact of lesion location on dysphagia incidence, pattern and complications in acute stroke. Part 1: dysphagia incidence, severity and aspiration. Eur J Neurol. (2015) 22:832–8. doi: 10.1111/ene.12670, PMID: 25677582

[17] Suntrup-Krueger S Kemmling A Warnecke T Hamacher C Oelenberg S Niederstadt T. The impact of lesion location on dysphagia incidence, pattern and complications in acute stroke. Part 2: oropharyngeal residue, swallow and cough response, and pneumonia. Eur J Neurol. (2017) 24:867–74. doi: 10.1111/ene.13307, PMID: 28449405

[18] Wilmskoetter J Martin-Harris B Pearson WG Jr Bonilha L Elm JJ Horn J. Differences in swallow physiology in patients with left and right hemispheric strokes. Physiol Behav. (2018) 194:144–52. doi: 10.1016/j.physbeh.2018.05.010, PMID: 29758228 PMC6070395

[19] Crisan D Shaban A Boehme A Dubin P Juengling J Schluter LA. Predictors of recovery of functional swallow after gastrostomy tube placement for dysphagia in stroke patients after inpatient rehabilitation: a pilot study. Ann Rehabil Med. (2014) 38:467–75. doi: 10.5535/arm.2014.38.4.467, PMID: 25229025 PMC4163586

[20] Li J Zhang J Li S Guo H Qin W Hu WL. Predictors of percutaneous endoscopic gastrostomy tube placement after stroke. Can J Neurol Sci. (2014) 41:24–8. doi: 10.1017/s0317167100016218, PMID: 24384333

[21] Dubin PH Boehme AK Siegler JE Shaban A Juengling J Albright KC. New model for predicting surgical feeding tube placement in patients with an acute stroke event. Stroke. (2013) 44:3232–4. doi: 10.1161/STROKEAHA.113.002402, PMID: 23963332 PMC3885340

[22] Otite FO Nene Y Sabra A Pfaff L Anikpezie N Akano EO. Percutaneous endoscopic gastrostomy usage in acute ischemic stroke: an analysis of trends in the United States from 2006 to 2022. Neurol Clin Pract. (2025) 15:e200495. doi: 10.1212/CPJ.0000000000200495, PMID: 40584639 PMC12204765

[23] Lorca-Puls DL Gajardo-Vidal A White J Seghier ML Leff AP Green DW. The impact of sample size on the reproducibility of voxel-based lesion-deficit mappings. Neuropsychologia. (2018) 115:101–11. doi: 10.1016/j.neuropsychologia.2018.03.014, PMID: 29550526 PMC6018568

[24] Austin PC. Statistical criteria for selecting the optimal number of untreated subjects matched to each treated subject when using many-to-one matching on the propensity score. Am J Epidemiol. (2010) 172:1092–7. doi: 10.1093/aje/kwq224, PMID: 20802241 PMC2962254

